# Differences between the effects of plant species and compartments on microbiome composition in two halophyte *Suaeda* species

**DOI:** 10.1080/21655979.2022.2076009

**Published:** 2022-05-20

**Authors:** Mu Peng, Chao Wang, Zhiyong Wang, Xiufang Huang, Fangzhen Zhou, Shaopeng Yan, Xiaopeng Liu

**Affiliations:** a College of Biological Science and Technology, Hubei Minzu University, Hubei, China; b College of Life Sciences, Northeast Forestry University, Harbin, China; c Zibo Academy of Agricultural Sciences, Zibo, China

**Keywords:** Halophyte, *Suaeda*, bacterial diversity, plant compartments, network analysis

## Abstract

Root-related or endophytic microbes in halophytes play an important role in adaptation to extreme saline environments. However, there have been few comparisons of microbial distribution patterns in different tissues associated with halophytes. Here, we analyzed the bacterial communities and distribution patterns of the rhizospheres and tissue endosphere in two *Suaeda* species (*S. salsa* and *S. corniculata* Bunge) using the 16S rRNA gene sequencing. The results showed that the bacterial abundance and diversity in the rhizosphere were significantly higher than that of endophytic, but lower than that of bulk soil. Microbial-diversity analysis showed that the dominant phyla of all samples were *Proteobacteria, Actinobacteria, Bacteroidetes, Acidobacteria* and *Firmicutes*, among which *Proteobacteria* were extremely abundant in all the tissue endosphere. Heatmap and Linear discriminant analysis Effect Size (LEfSe) results showed that there were notable differences in microbial community composition related to plant compartments. Different networks based on plant compartments exhibited distinct topological features. Additionally, the bulk soil and rhizosphere networks were more complex and showed higher centrality and connectedness than the three endosphere networks. These results strongly suggested that plant compartments, and not species, affect microbiome composition.

## Graphical abstract

**Figure:**
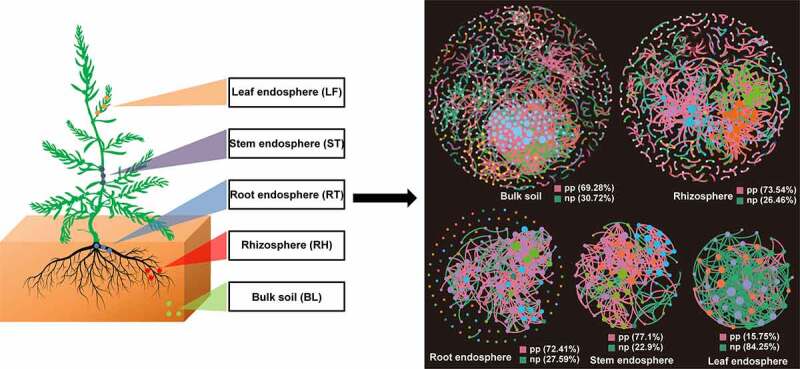

## Highlights

- There were notable differences in bacterial diversity related to plant compartments.
- The bulk soil and rhizosphere networks showed higher centrality and connectedness.
- Plant compartments, and not species, affect microbiome composition.

## 1. Introduction

Rhizosphere soil attached to the plant root surface plays an important role in plant health and soil fertility [1]. Vigorous roots systems secrete a variety of organic compounds to stimulate the growth of rhizosphere microorganisms [2,3]. Thus, the microbial community of the soil-root interface (rhizosphere microenvironment) is established. Because of the differences in root secretion and rhizosphere deposition, plant species could also affect the structure and functional diversity of rhizosphere microorganisms [4–6]. Microbial communities have been analyzed by culture and non-culture methods, and the results showed that the rhizosphere had a significant influence on the microbial composition [7]. In the natural soil environment, the growth of soil microbes is usually nutrient-limited, whereas root secretions such as organic acids, sugars and amino acids could stimulate bacterial growth and extracellular enzyme activity. In this way, root secretions affect the biogeochemical cycling of carbon, nitrogen, phosphorus and sulfur in the soil [8]. Therefore, it is important to understand the effect of plant roots on the composition of microbial communities in the rhizosphere and soil. Plant roots release 17% of photosynthetic products, and most of them can be used for the growth and reproduction of microbes [9]. However, most of the relevant studies were carried out in the non-rhizosphere soil of agricultural crops, so there are few reports on the influence of the root system on the soil microbial community structure of halophytes [10,11].

Endophytic bacteria are a group of bacteria living in plant cells, or in intercellular or vascular systems. They can help host plants by promoting plant growth or providing biological control of plant diseases [12]. Many studies have confirmed that endophytic bacteria enhance growth and alleviate salinity stress in halophyte species using special reference to osmotic and ionic stress management [13,14]. [15], pointed out that most plant species, including monocotyledonous and dicotyledonous plants, as well as woody and herbaceous species, contain a variety of endophytic bacteria. A better understanding of endophytic bacteria may help to elucidate their function and potential role in improving plant performance.

Halophytes, such as *Suaeda* species, play an important role in carbon sequestration, nutrient mineralization, nutrient cycling and ecological remediation of the microenvironment [16,17]. There are some reports on the rhizosphere microbial community and endophytic bacterial community of halophytes [18–20]. However, those studies focused on only one or unrelated halophyte species (different family or genus). Whether the rhizosphere environment and endophytic bacteria of closely related halophytes have unique microbial communities or species specificity remains to be determined.

This study raises two questions: (1) what are the differences between bacterial communities in the rhizosphere and different plant compartments of *Suaeda*? (2) whether different habitats, such as a root environment and different tissues, may affect the structure of a bacterial community. To solve these issues, 16S rDNA high-throughput sequencing technology was used to analyze the structure and diversity of bacterial communities in the rhizosphere, leaves, stems and roots of different *Suaeda* species. These results provide a basis for further study of the relationship between the rhizosphere and endophytic bacteria in different plant organs.

## 2. Materials and methods

### 2.1 Sample collection and surface sterilization

Sampling sites were located in Daqing, Heilongjiang Province, China, at the latitude of 46°01’~47°01ʹN, longitude of 124°53’~125°55ʹE. The area is characterized by a temperate continental semi-arid monsoon climate with a mean annual air temperature of 3.2°C. The zonal soil is mainly soda saline-alkali earth with the pH of 9–10. More detailed information about the soil is listed in Table S1.

*Suaeda* species are the dominant species in this zone. Three plots (10 m × 20 m each) were selected with three healthy plants of each species [*S. salsa* (SS), *S. corniculata* Bunge (SC)] (three plots × three plants/species = nine plants/species). The bulk soils 10 cm away from the plant were collected at a depth of 0–10 cm using a 10-spot sampling method and mixed. The collected samples included leaves (LF), stems (ST), roots (RE), rhizosphere soil (RH), and bulk soil (BL) from this sampling site. All soil samples had the solid salt crusts and litter layer removed, were placed in aseptic bags, and then transported back to the lab. The soil adhered to the root surface was rhizosphere soil. The three complete plants from the same plot (LF, ST, and RE) were mixed as one sample and cut into pieces (approximately 3 cm). All tissue samples were washed with 10 mM PBS buffer and then surface-sterilized with 5% NaClO for 5 min, and washed with sterile water 5 times. After the sterilization process, 100 μL of water used in the final rinse was dripped into tryptic soy agar medium and incubated at 28°C for 3 days. If no bacteria colony was detected by a culture-dependent sterility test, then the tissue samples could be used for further analysis.

### 2.2 Microbial genomic DNA extraction, PCR and sequencing

All tissue samples were frozen and ground rapidly into a powder in a mortar and then transferred to a bead tube. For the rhizosphere and bulk samples, 0.1 g fresh soil was used for microbial DNA extraction (Power Soil DNA Isolation Kit, Qiagen) according to the manufacturer’s instructions. The extracted DNA was detected by 2% agarose gel electrophoresis, and the purity of DNA was quantitatively analyzed with NanoDrop. Genomic DNA was used to amplify 16S rRNA gene with the specific primers (341 F/806 R). The PCR conditions, sequencing and construction of bacterial 16S rRNA gene were performed with the Illumina MiSeq platform (Sangon Biotech, Shanghai) following our previous reports [21,22].

### 2.3 Bioinformatics analysis process

Raw data was preprocessed using QIIME data analysis package [23], then assembled and filtered to ensure the most efficient data clustering into Operational Taxonomic Units (OTU) with 97% sequence similarity as the cutoff point using USEARCH [24]. OTUs classified as chimeras, chloroplast or mitochondria were removed using the MOTHUR program [25,26]. After obtaining filtered OTUs, species annotation, α- and β-diversity analysis were carried out according to the analysis process [27]. In addition, a heatmap and Venn diagrams were generated with an R-package [28]. Furthermore, we used Linear discriminant analysis Effect Size (LEfSe) software (v1.0) to identify differentially abundant families among samples for biomarker discovery [29]. In order to compare the different samples, three complementary non-parametric statistical methods (Adonis, ANOSIM and MRPP) were used to determine the overall differences in bacterial communities [28].

Based on the OTU table, we constructed the microbial community ecological network using a phylogeny molecular ecological network analysis pipeline (pMENA) (http://ieg4.rccc.ou.edu/mena/) [30]. According to the topological role of the microbial community in the ecological network, some potential keystones were identified. The topology type of each node was determined based on within-model connectivity (*Zi*) and among-module connectivity (*Pi*). According to the values of *Zi* and *Pi*, four categories were classified, namely, peripherals (*Zi* < 2.5, *Pi* < 0.62), connector (*Zi* < 2.5, *Pi* > 0.62), module hubs (*Zi* > 2.5, *Pi* < 0.62) and network hubs (*Zi* > 2.5, *Pi* > 0.62). The last three categories played an important role in network topology and were considered as key taxa [31].

### 2.4 Data access

All the bacterial raw sequences have been deposited to GenBank Short Read Archive (PRJN593778).

## 3. Results

### 3.1 Diversity analysis

**Figure 1.**
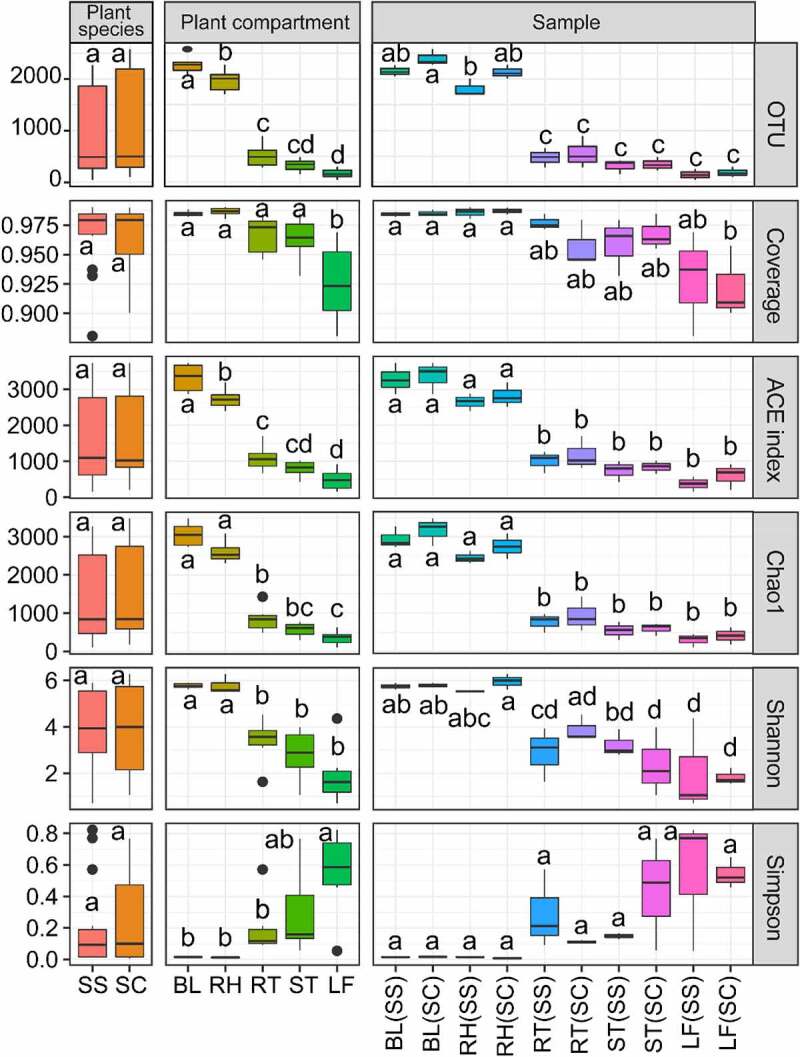
Alpha-diversity indices for the 16S rRNA gene sequence in different samples.

Because of the very different numbers of clean reads between rhizosphere and tissue endosphere, those two types of samples were compared at different sequencing depths by subsampling the first 146 reads and 2142 reads for tissue endosphere and rhizosphere, respectively. The number of OTUs was the highest in the BL, followed by RH, RT and ST, and the lowest in LF (Figure 1). ANOVA results showed that there was no significant difference in the number of OTUs among different plant species and that the number of OTUs in soil bacteria was significantly higher than that of tissue endophytic bacteria. ACE and Chao indexes in BL(SS) and BL(SC) samples were the highest value, indicating that the microbial community in the bulk control soil was the most abundant, whereas the lowest values were in the root samples (Figure 1). A similar result was found in Shannon index and a reverse trend was detected in Simpson index. In all samples, the value of the Coverage index was greater than 0.94, indicating that the sequencing results covered almost all bacteria in the samples. The alpha-diversity results showed that the bacterial communities were significantly different in plant compartments. Those results showed that the rhizosphere and bulk soil samples were significantly higher than tissue samples, while bacterial diversity in the two kinds of *Suaeda* species did not reach the significant level.

SS: *Suaeda salsa*, SC: *S. corniculata*; BL: bulk soil, RH: rhizosphere soil; BL(SS), RH(SS), RT(SS), ST(SS) and LF(SS) indicate bulk soil, rhizosphere soil, root, stem and leaf under *S. salsa*, respectively; BL(SC), RH(SC), RT(SC), ST(SC) and LF(SC) represent bulk soil, rhizosphere soil, root, stem and leaf under *S. corniculata*, respectively. Different letters indicate significant difference between means, lower case indicates *P* < 0.05.

**Figure 2.**
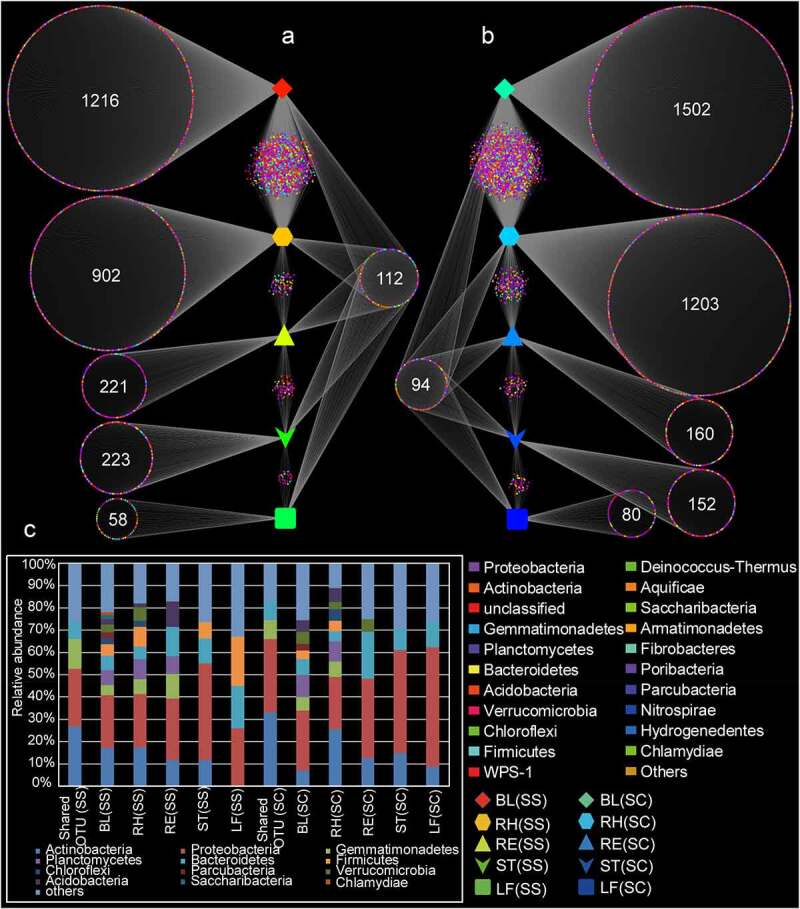
Venn diagram analysis showing the number of unique and shared OTU in different samples. BL(SS), RH(SS), RT(SS), ST(SS) and LF(SS) indicate bulk soil, rhizosphere soil, root, stem and leaf under *S. salsa*, respectively; BL(SC), RH(SC), RT(SC), ST(SC) and LF(SC) represent bulk soil, rhizosphere soil, root, stem and leaf under *S. corniculata*, respectively. A: the number of shared and unique OTUs in the different plant compartments of *S. salsa* species; B: the number of shared and unique OTUs in the different plant compartments of *S. corniculata* species; C: the relative abundance of shared and unique OTUs.

According to the Venn diagram analysis, a total of 112 and 94 shared OTUs were found in the samples of *S. salsa* and *S. corniculata*, respectively, with the majority of OTUs assigned to *Proteobacteria* and *Actinobacteria* (Figure 2A and 2C). The numbers of OTUs exclusive to the LF(SS), ST(SS), RE(SS), RH(SS), and BL(SS) samples were 58, 223, 221, 902 and 1216, respectively, with most OTUs belonging to *Proteobacteria*. Similar results were found in *S. corniculata* samples (Figure 2B and 2C), implying habitat-specific patterns. To sum up, the number and species of the specific and shared OTUs were similar in the same plant compartments of two different halophytes.

### 3.2 Taxonomic analysis at different levels

**Figure 3.**
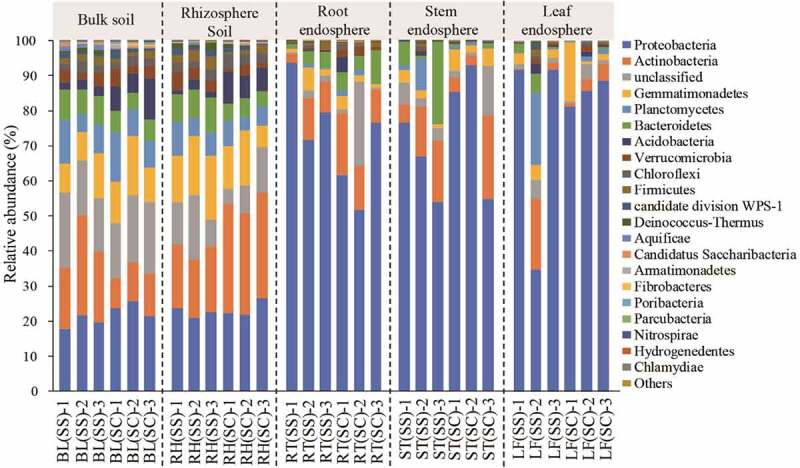
The relative abundance of bacteria at the phylum level in the different samples.

A total of 36 phyla were identified including some unclassified bacteria (Table S2). In Figure 3, the predominant phyla included *Proteobacteria, Actinobacteria, Gemmatimonadetes, Planctomycetes, Bacteroidetes, Acidobacteria, Verrucomicrobia, Chloroflexi, Firmicutes* and *WPS-1*, which could be detected in all samples with varied values. The relative abundances of *Proteobacteria* and *Actinobacteria* were the highest in all samples, accounting for 19.65% – 85.14% and 3.02% – 30.08% of the total sequences, respectively. Interestingly, the average abundance of *Proteobacteria* in the tissue samples was more than 60%, which was significantly higher than that in the rhizosphere and bulk soil (*P*< 0.01). The relative abundance of *Gemmatimonadetes* was about 10% in the soil samples, which was much higher than that in the other samples.

BL(SS), RH(SS), RT(SS), ST(SS) and LF(SS) indicate bulk soil, rhizosphere soil, root, stem and leaf under *S. salsa*, respectively; BL(SC), RH(SC), RT(SC), ST(SC) and LF(SC) represent bulk soil, rhizosphere soil, root, stem and leaf under *S. corniculata*, respectively.

**Figure 4.**
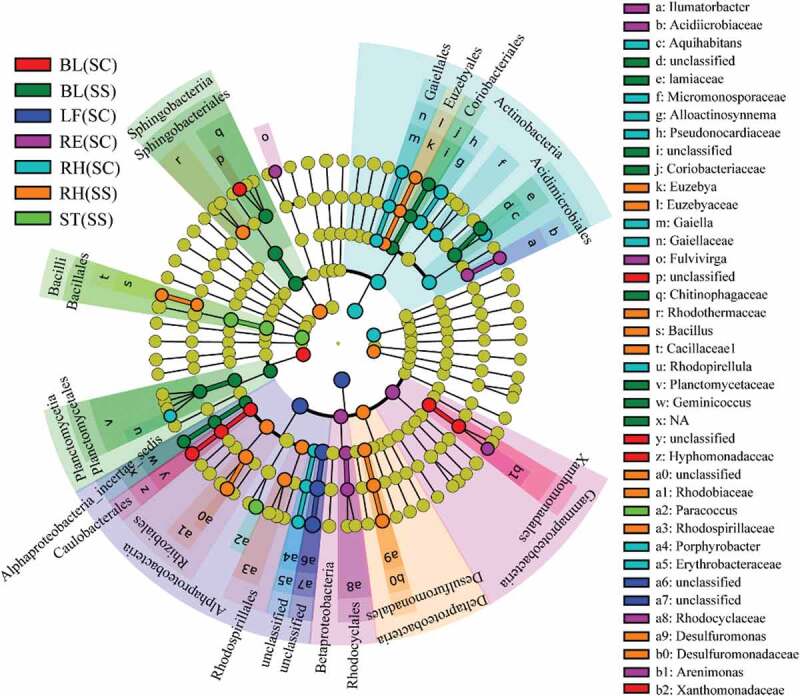
LEfSe analysis indicating the phylogenetic distribution of microbial lineages associated with the different samples. Differences are represented in the color of the most abundant class. Circles represent phylogenetic levels from domain to genus inside out. RE, root endosphere; RH, rhizosphere; BL, bulk control soil; LF: leaf; RT: root; ST: stem; SS, *S. salsa*; SC: *S. corniculata* Bunge.

LEfSe was applied to find the significantly different taxa abundances among the different plant compartments (Figure 4). Based on the family level, *Planctomycetaceae, Alphaproteobacteria, Lamiaceae, Coriobacteriaceae* and *Acidimicrobiaceae* were predominantly distributed in the BL(SS) samples. *Hyphomonadaceae* and *Xanthomonadaceae* were mainly distributed in the BL (SC) samples. *Rhodocyclaceae* and *Acidimicrobiaceae* were more abundant in the RE (SC) samples. *Pseudonocardiaceae, Gaiellaceae, Micromonosporaceae* and *Erythrobacteraceae* were higher in the RH (SC) samples. *Rhodothermaceae, Rhodobiaceae,Rhodospirillaceae, Desulfuromonadaceae, Euzebya* and *Bacillaceae* were more abundant in the RH(SS) samples. A total of 608 genera were identified. Through clustering of the top 50 genera, we found that the distribution of species was significantly different in different samples (Fig.S1 and Table S3). Heatmap and LEfSe results showed that there were notable differences in microbial community composition related to plant compartments. In addition, three complementary non-parametric multivariate statistical tests (Adonis, ANOSIM and MRPP) further showed that the bacterial communities in different plant compartments were significantly different (*P* < 0.05), expect for bulk vs rhizosphere soils, roots vs leaves, which may be caused by sequencing depth (Table 1). Interestingly, statistical results indicated that bacterial communities were not different among plant species and sample types.

**Table 1.** Significance tests of the effects of on the bacterial community structure with three different statistical approaches

	Adonis		ANOSIM		MRPP	
	*F*	*P*	*R*	*P*	*δ*	*P*
Total	4.436	**0.001**	0.727	**0.001**	0.559	**0.001**
Plant species	1.425	0.141	0.037	0.161	0.812	0.113
Bulk soil	3.546	0.1	0.926	0.1	0.550	0.1
Rhizosphere soil	2.49	0.1	0.667	0.1	0.700	0.1
Root	1.722	0.1	0.333	0.2	0.663	0.1
Stem	1.257	0.5	0.037	0.5	0.648	0.3
Leaf	0.891	0.6	−0.148	1	0.484	1
Above ground: under ground*	8.381	**0.001**	0.606	**0.001**	0.724	**0.001**
Soil: tissue	16.117	**0.001**	0.993	**0.001**	0.666	**0.001**
Bulk soil: Rhizosphere soil	1.355	0.121	0.096	0.141	0.625	0.123
Bulk soil: root	9.096	**0.003**	0.998	**0.003**	0.607	**0.002**
Rhizosphere soil: root	6.068	**0.002**	0.985	**0.004**	0.682	**0.002**
Rhizosphere soil: stem	7.148	**0.002**	1	**0.001**	0.674	**0.001**
Rhizosphere soil: leaf	9.809	**0.002**	1	**0.003**	0.592	**0.003**
Root: stem	1.489	0.084	0.109	0.127	0.656	0.068
Root: leaf	5.038	**0.005**	0.556	**0.008**	0.574	**0.004**
Stem: leaf	3.013	**0.009**	0.270	**0.017**	0.566	**0.007**

*Above ground: stem and leaf, under ground: root, rhizosphere soil and bulk soil. Bold values represent a signifcant difference (P < 0.05).

### 3.3 Network analysis

**Figure 5.**
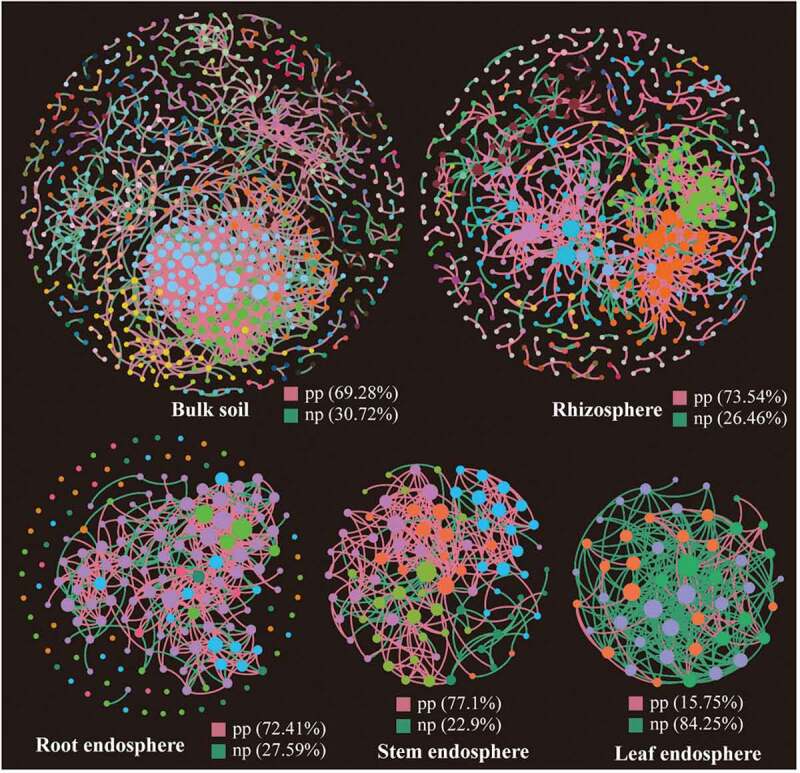
Phylogenetic molecular ecological networks based on different *Suaeda* species microbiomes and the topological roles of nodes. A node represents an OTU, and its color represents its module. The green and red lines indicate negative and positive interaction, respectively. pp: positive interaction; np: negative interaction.

The topological properties of molecular ecological networks (MENs) of different *Suaeda* species and their associated random MENs were listed in Table 2. According to the total number of nodes and links, networks based on bulk and rhizosphere soils showed more complexity than the other three tissue endosphere networks. Interestingly, the bacterial communities in leaf endosphere networks tended to have negative relationship among nodes, accounting for 84.25% of the potential interactions (Figure 5). While in the other four networks, the positive correlations accounted for 69.28%-77.1%.

**Table 2.** Topological properties of molecular ecological networks (MENs) of different *Suaeda* species and their associated random MENs

Network name	Topological properties	BL	RH	RT	ST	LF
Empirical networks	Similarity threshold	0.98	0.98	0.80	0.75	0.70
	Total nodes	705	510	109	86	49
	Total links	1465	756	395	262	254
	Average degree (*avgK*)	4.16	4.39	7.25	6.09	10.37
	Betweenness	0.16	0.16	0.13	0.24	0.13
	Degree Centrality	0.08	0.06	0.16	0.13	0.34
	Average path (GD)	9.32	7.12	3.36	3.31	2.05
	Average clustering coefficient (*avgCC*)	0.23	0.25	0.38	0.39	0.29
	Modularity	0.725	0.71	0.55	0.53	0.22
Random networks	Modularity	0.49 ± 0.01	0.63 ± 0.01	0.30 ± 0.01	0.26 ± 0.01	0.18 ± 0.01
	Average path (GD)	4.07 ± 0.04	4.76 ± 0.07	2.64 ± 0.03	2.34 ± 0.01	1.94 ± 0.02
	Average clustering coefficient (*avgCC*)	0.02 ± 0.00	0.01 ± 0.00	0.10 ± 0.01	0.014 ± 0.01	0.29 ± 0.02

BL: network based on bulk soil of different *Suaeda* species.

RH: network based on rhizosphere soil of different *Suaeda* species.

RT: network based on root endosphere of different *Suaeda* species.

ST: network based on stem endosphere of different *Suaeda* species.

LF: network based on leaf endosphere of different *Suaeda* species.

**Figure 6.**
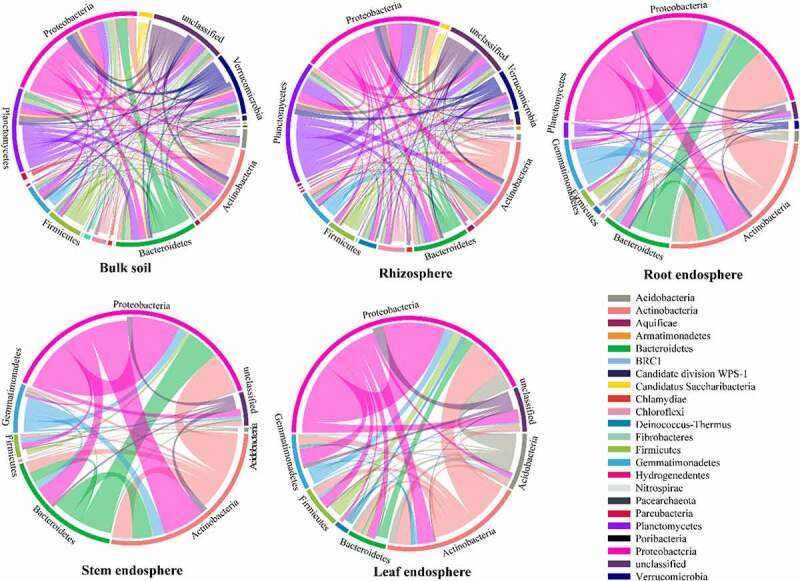
Circos plots showing the distribution of links among interacting phyla in different networks.

The composition of nodes and links in each network were different, and most of the network connections came from the links between *Proteobacteria* and other bacteria (Figure 6). These differences in the composition of different network connections were consistent with the topological characteristics of their network structure. In addition, the composition of nodes and links showed different trends in the networks (Figure 6). For example, in two soil bacterial networks, the relative abundance of *Actinobacteria* was higher than *Planctomycetes*, whereas *Planctomycetes* accounted for more links than *Actinobacteria*. In the three tissue endophytic bacterial networks, the node ratio of *Acidobacteria* was lower than that of *Bacteroidetes*, while in the endophytic bacterial network of leaves, the link ratio of *Acidobacteria* was much higher than that of *Bacteroidetes*. Therefore, there were more interactions with *Acidobacteria* than with *Bacteroidetes*.

The band between two segments represents the network connection among phylum, and the width of indicates the number of connection.

Subsequently, we identified the potential keystone taxa in the networks (peripheral nodes, connectors, module hubs, and network hubs) (Fig.S2A). The result showed that most (96.37%) OTUs were peripheral nodes (*Pi* < 0.62, *Zi* < 2.5). Among them, 78.41% had no connection with other modules (*Pi* = 0). However, network hubs, the most critical group, (*Pi* > 0.62, *Zi* > 2.5) were not detected in these networks. A total of 53 key groups (OTU) were detected in the networks, including 32 connectors and 21 module hubs, mainly belonged to *Proteobacteria* (40.91%), *Gemmatimonadetes* (11.36%) and *Planctomycetes* (11.36%) (Fig.S2B). The number of module hubs from the bulk soil to tissue endosphere showed a decreasing trend, which was in line with the decreasing trend of the number of modules in the corresponding network. In contrast, the number of connectors increased gradually, indicating that when the microbial community structure was relatively small, the interaction between species increased to cope with complex and changeable environmental factors. These results suggested that plant compartments significantly changed the network structure and topological properties of individual OTUs and key microbial populations. It is worth noting that the relative abundance of most module hubs (83.33%) and connectors (85.71%) was very low (< 1%), revealing the potentially important role of rare groups in the bacterial community (Fig.S2B).

## 4. Discussion

### 4.1 Plant compartments affect community structures

In this study, we detected the endophytic and rhizosphere bacterial diversity of two halophyte *Suaeda* species, showing that there were obviously different community structures in different plant compartments and that the bacterial diversity of the bulk and rhizosphere soils was higher than that of tissue endosphere (Table 1, Figures 1 and 2). Similar results were also found by [20],and [11]. Compared with the bulk soil, root systems have been regarded as a more stable ecological niche for bacteria [32,33], and, correspondingly, less bacterial diversity in roots has been observed compared to the rhizosphere and bulk soils. The bacteria in soil is mainly affected by its surrounding soil characteristics, whereas bacteria in the rhizosphere and roots might be greatly influenced by root secretions, soil, and plant growth stage, thus suggesting that microbial diversity decreases gradually from bulk soil to root system [34]. The root exudates, slime and exfoliated cells secreted by root cap, along with rhizosphere sediments of decaying roots together form a highly active transition zone in rhizosphere, providing an appropriate ecological niche for the growth of soil bacterial communities. On the other hand, microorganisms must overcome the plant immune defense mechanisms to survive and colonize the plant tissue, resulting in the endophytic bacteria being less complex than its surface bacteria [35].

Since most endogenous bacteria come from the soil and have a certain similarity with soil microbial communities, the diversity of endogenous bacteria in root systems is higher than that in leaf and stem tissues, which may be attributed to the fact that the root system is the main place where plants interact with soil [36]. At the same time, the low diversity of endophytic bacteria in leaves may be related to the low diversity of the phyllosphere [37] since some endogenous bacteria in leaves may come from phyllosphere [38]. The stem is less exposed to changing physical and chemical conditions, such as temperature, humidity, ultraviolet radiation and nutrients in the apoplast. Also, the starch, sugar and other nutrients from xylem sap in such a nutrient-rich ecosystem in the stems favor a greater number and variety of bacteria. However, the leaf-associated microenvironment is generally considered to be associated with limited nutrient supply and rapid changes in environmental conditions, so the leaf-associated bacterial community structure is highly heterogeneous [32]. Therefore, in our study, it is understandable that the stem-related bacterial diversity is higher than that of leaves.

Researchers in *Arabidopsis thaliana* showed that soil type and host genotype affected the root microflora to some extent [5], and they also found that the bacterial communities varied markedly in different development stages and plant genotypes. [39], also reported significant differences in the bacterial community in the roots of four sugarcane varieties. However, in the present study, endophyte and soil microbes were seen to be significantly different in halophytes, and these differences mainly depended on plant compartments, not plant species (Table 1). We found that each sample harbored their unique bacteria. *Proteobacteria* sequences were common in all sample types, but the most abundant in *S. salsa* was *γ-Proteobacteria*, whereas in *S. corniculata* it was *β-Proteobacteria*. The presence of *Sphingomonas* in the stem of *S. corniculata* was significantly higher than in the other samples. *Sphingomonas* has potentially beneficial effects on plant growth and health [40], and some strains of *Sphingomonas* isolated from various environments and plant samples have protective effects against plant pathogens [41]. As a common soil and aquatic bacterium, *Roseomonas* has mostly been isolated from clinical patient specimens [42,43]. It was identified from plant tissues, mainly in the stem of *S. corniculata*, for the first time in our study. The relative abundance of *Euzebya* was the highest in the rhizosphere soil of *S. corniculata*. This genus is mostly a saccharide bacteria, which can acquire carbon and energy through the hydrolysis of carbohydrates and participate in the carbon cycle in the soil ecosystem [44]. It also has a putative ability as a biocatalyst to degrade various harmful compounds from the production of fossil fuels and bioactive steroids [45].

### 4.2 Core plant microbiome

In microbial communities, the discovery of core microbes was important for understanding the stability and consistency of complex microbial networks [46]. A core microbial community consists of two or more members related to habitats [47]. By defining shared OTUs from similar habitats in overlapping areas of Venn diagrams, we found that there were 112 and 94 OTUs in *S. salsa* and *S. corniculata* samples, respectively (Figure 2), among which the dominant phyla were mainly *Proteobacteria* and *Actinobacteria*. These common groups were regarded as the core microorganisms in *Suaeda* species, and they were defined as the key groups shared among the microbial communities. The predominance of these phyla was consistent with their rapid growth characteristics and their ability to use a variety of root carbon matrix from plants [48]. These dominant bacteria were also found in other studies, which assessed the diversity of rhizosphere and bulk soils of different halophytes [11]. In addition, these core microorganisms might play an important role in the function and stability of microflora [49]. [46], suggest that core OTUs are critical to elucidating the ecology of microbial communities, and microbes associated with specific habitats might be critical to community function.

### 4.3 Bacterial networks

Network analysis could help us to identify microbial associations, to better understand interactions within functional bacterial communities, and to assess the possible topological roles of each taxon in the network [50–52]. The network analysis in our study showed distinct structural characteristics in different plant compartments, and the niche differentiation of these compartments in significantly different microenvironments could explain the difference in topological traits. The niche differentiation of endophytic bacterial community is low due to the limitation of host plant space and nutrients, which might lead to the intensification of competition. Similar niche differentiation also exists among soil microbial communities, which may be the result of the combination of root deposition and fine regulation of host genotype dependence, and this differentiation then leads to a selection of specific bacteria combinations [53,54]. The correlation among OTUs in the leaf endosphere network was mainly negative, while all of the other four networks were positive (Figure 5), indicating that most bacterial groups had the potential for extensive cooperation and symbiosis in their respective microenvironments. However, negative feedback between nodes in the network tends to stabilize the processes, while positive feedback enhances the changes of the ecosystem and destroys the current situation of network structure [55]. Therefore, the endophytic bacteria in leaves might be more resistant to environmental disturbance than the soil bacteria.

Many studies demonstrate that hubs or connectors can play a vital role as keystone groups in stabilizing ecosystems [31,56]. However, they do not always exist together [57]. Different numbers of module hubs and connectors were detected in the five networks of *Suaeda* species. The highest number of module hubs were detected in the bulk soil network, indicating that this network was more orderly than other networks, as more module hubs can maintain and stabilize the orderliness of microbial community structure [58,59]. The highest number of connectors were found in our stem endosphere network. More connectors could organize a series of modules into a complete community, thus improving the efficiency of energy metabolism, nutrient cycling and material transformation in the environment [52]. In addition, the bacterial communities based on the five ecological networks exhibit a highly modular architecture (Table 2). One possible explanation for this structural characteristic is the absence of keystone taxa, i.e., no network hub is detected in any network structures [60]. Compared with other taxa, network hubs play a disproportionately important role in maintaining network structure, so the disappearance of these key taxa may divide the network into more modules [31]. Under certain conditions, due to niche differentiation, modules can be regarded as functional units [61]. The modularization value of the soil bacterial network was much higher than that of endophytic bacterial network, which may be because the soil niche differentiation level is higher than that of plant endosphere. Due to the unique organizational structure in plant tissue, available niches are limited, resulting in more clustered network structures.

## 5. Conclusion

The rhizosphere and endophytic bacterial community and diversity of two *Suaeda* species were compared using 16S rRNA gene sequencing. In all the samples, the dominant bacteria mainly included *Proteobacteria* and *Actinobacteria*, indicating the importance of these bacteria in the microecology of *Suaeda* species. Heatmap, LefSe and non-parametric statistical results showed that the distribution of bacteria in different plant compartments significantly varied, suggesting that compartments affect microbiome composition, not species. Different networks based on plant compartments exhibited distinct topological features. Additionally, the bulk soil and rhizosphere networks were more complex and showed higher centrality and connectedness than the three endosphere networks.

## Supplementary Material

Supplemental Material

## Data Availability

All data included in this study are available upon request by contact with the corresponding author (https://dataview.ncbi.nlm.nih.gov/object/PRJNA593778).
