# Supplementary material for: Differences between the effects of plant species and compartments on microbiome composition in two halophyte Suaeda species
Source: Bioengineered. 2022 May 20;13(5):12475–88. doi: 10.1080/21655979.2022.2076009 (PMC9275862; doi:10.1080/21655979.2022.2076009)
Supplement: Supplemental Material [file KBIE_A_2076009_SM0454.zip › supplementary/Fig S1.docx]

Fig. S1 Heatmap of bacterial distribution of the top 50 abundant genera in the different samples. RE, root endosphere; RH, rhizosphere; BL, bulk control soil; LF: leaf; RT: root; ST: stem; SS, *S. salsa*; SC: *S. corniculata* Bunge.
