# Supplementary material for: Differences between the effects of plant species and compartments on microbiome composition in two halophyte Suaeda species
Source: Bioengineered. 2022 May 20;13(5):12475–88. doi: 10.1080/21655979.2022.2076009 (PMC9275862; doi:10.1080/21655979.2022.2076009)
Supplement: Supplemental Material [file KBIE_A_2076009_SM0454.zip › supplementary/Fig S2.docx]

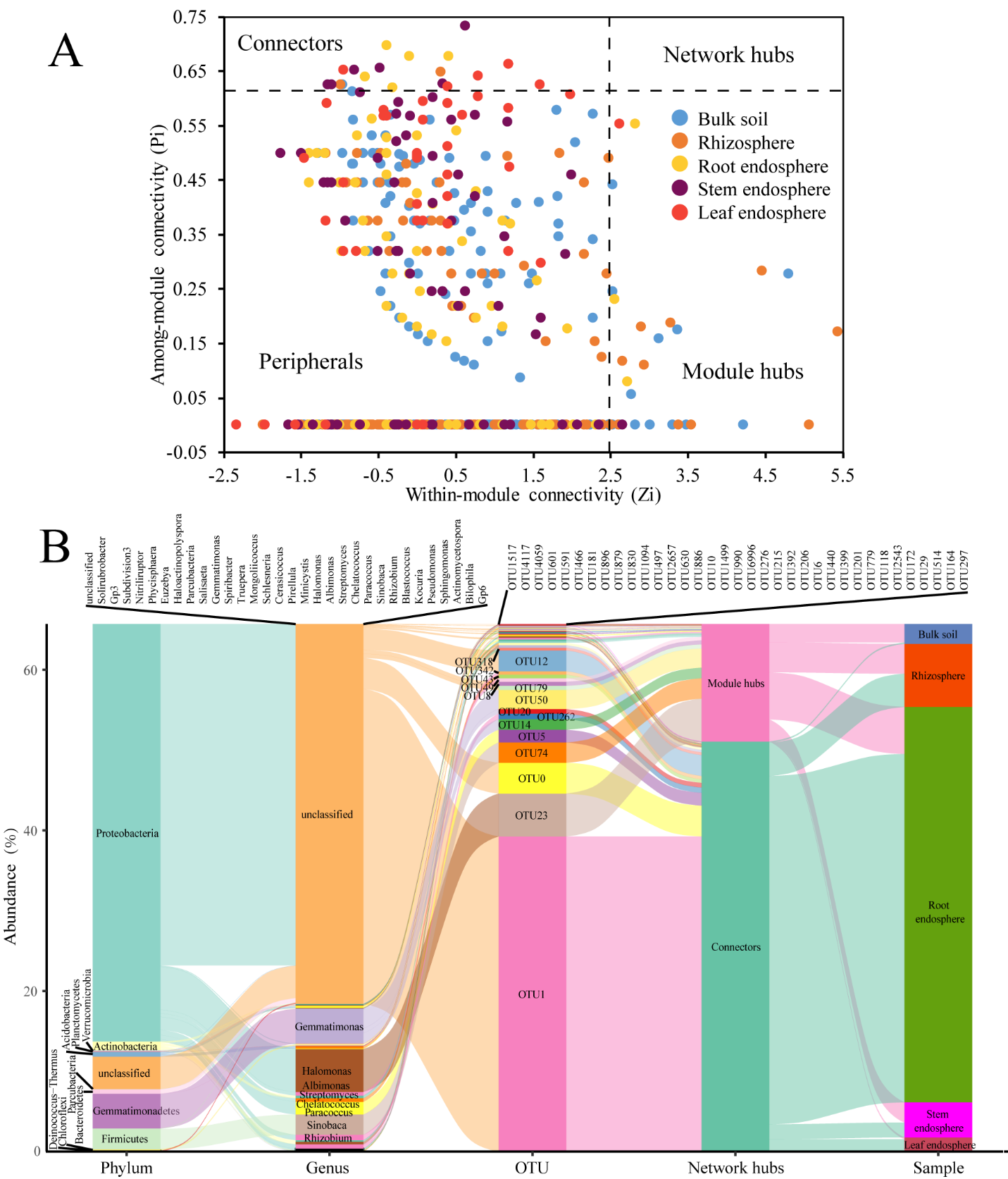


Fig. S2 Putative keystone taxa in the different networks based on Pi and Zi and its system classification levels and relative abundance in different networks

According to *Pi* and *Zi* values, the nodes are divided into peripherals, connectors, module hubs and network hubs with their possible ecological functions. The solid circle represents an OTU and it color shows different network.
