# Supplementary material for: Differences between the effects of plant species and compartments on microbiome composition in two halophyte Suaeda species
Source: Bioengineered. 2022 May 20;13(5):12475–88. doi: 10.1080/21655979.2022.2076009 (PMC9275862; doi:10.1080/21655979.2022.2076009)
Supplement: Supplemental Material [file KBIE_A_2076009_SM0454.zip › supplementary/Table S1.docx]

Table S1 Differences of physicochemical properties between rhizosphere and bulk soils

|  | BL(SS) | RH(SS) | BL(SC) | RH(SC) |
| --- | --- | --- | --- | --- |
| pH | 10.39±0.10 aA | 9.15±0.15 bB | 10.29±0.02 aA | 9.04±0.26 bB |
| EC (dS/m) | 1272.21±81.25 aA | 1243.37±97.68 aA | 1312.02±140.42 aA | 1307.35±76.46 aA |
| Water content (%) | 13.50±0.54 bC | 15.96±0.85 aAB | 13.74±0.60 bBC | 16.69±0.57 aA |
| Total P (mg/kg) | 0.03±0.00 bB | 0.13±0.00 aA | 0.03±0.00 bB | 0.13±0.01 aA |
| Total N (g/kg) | 0.06±0.00 bB | 0.09±0.00 aA | 0.06±0.01 bB | 0.09±0.00 aA |
| Organic matter (mg/kg) | 0.94±0.22 bB | 1.67±0.04 bAB | 1.13±0.13 bB | 2.53±0.50 aA |

All statistical analysis based on SPSS software. Lowercase letters stand for significant difference at 5% level, uppercase letters stand for significant difference at 1% level.

RH, rhizosphere; BL, bulk control soil; LF: leaf; RT: root; ST: stem; SS, *S. salsa*; SC, *S. corniculata*.
